# A Case of Delirium Tremens Successfully Treated by Large Doses of Opium

**Published:** 1834-10-01

**Authors:** C. J. Roberts

**Affiliations:** Physician to the Welsh Charity, and Infant Orphan Asylum.


*A Case of Delirium Tremens successfully treated by large Doses of Opium.* By C. J. Roberts, M.D., Physician to the Welsh Charity, and Infant Orphan Asylum.

Notwithstanding the progress of Temperance Societies, and in despite of threatened parliamentary enactment, drunkenness would seem still to hold on its course, with rather increased than abated vigour; and most probably will so remain, until the world shall become more enlightened and better acquainted with the mischief it produces, both at the time, and for the remainder of a person's existence. I shall not recapitulate the various diseases which are engendered by the baneful habit of drinking, but intend merely to describe one, which, however much it may be supposed to be known and well understood, still, like all other maladies, has its varieties, which call for corresponding alterations in the method of treatment. The complaint I mean is Delirium Tremens, so called from two very prominent symptoms which always mark its progress,—the delirium or aberration of mind, and the tremulousness of the body, extremities, and tongue. This disease would seem to have escaped observation, until its peculiarities were detailed by Dr. Pearson, practising at Newcastle-upon-Tyne, about the year 1801, who first gave an accurate account of it, in a small pamphlet, which he then published. In this he shewed the necessity of quieting the irritation of the brain, produced by the immoderate use of fermented liquors, by other means than those of counter-irritation and depletion. Dr. Pearson details 93 cases, treated by the soothing plan, which all recovered; and then mentions others, who were depleted and managed antiphlogistically, and which terminated fatally. Dr. Sutton, of Greenwich, and the late Dr. Armstrong, have each demonstrated the same facts, and that so clearly, that I thought this method was generally allowed to be past denial the only one to be used in this complaint. Some practitioners I knew there were, who, having peculiar views of cerebral pathology, pursued the depleting plan, that they might at least have the credit of consistency; yet these were so few, that I considered their opinions rather more in the light of an eccentricity, than as being prosecuted with any thing approaching a seriousness of intention. I was however very much surprised to find that a Dr. Ware had published a paper, in the Transactions of the Massachussetts Medical Society for 1831, in which he seriously advocates this method of treatment, and as strongly reprobates the use of opium; asserting that, in many instances in which he has seen it employed, although it might have given temporary relief, yet the patients uniformly sunk afterwards, from its specific effects upon the brain. These are his words: "So far from being beneficial, I believe there is ground to believe that the effect of opium, given during the paroxysm, is to increase the violence of the delirium, to produce a tendency to convulsions, to prevent the termination by a natural and salutary sleep, or to throw the patient into a state of coma, from which he does not awake." (P. 49.)

These assertions are so far diametrically opposite to all our received notions of opium, and its effects in this disease, that I was first startled, and afterwards more especially surprised, to find, in looking further, that this gentleman impugns the good faith of others who have described cases of delirium tremens, by stating that, during their progress, depletion in some form or another was had recourse to, and that it was to that, and not to the opium, that the cure was attributable: and he even goes further, by maintaining that, in many instances, the administration of opium produces paroxysms of delirium tremens, which would not have occurred, had the narcotic not been administered. He does not however bring forward any evidence to prove what he asserts, but closes his attack upon the use of opium, in this species of cerebral irritation, by acknowledging that he has no power of shewing its deleterious tendency: "I do not mean to say that I have evidence to shew that opium is in all cases injurious in the diseases of the intemperate, or that it should never be employed."

During the fourteen years I have been in practice in London, I have treated many cases of this disease, and I only know of two persons who have escaped with life who were depleted at the commencement of the attack; and I have very seldom seen those who have suffered from epileptic paroxysms during any part of the progress of the disorder ever recover. During the time I was physician to the General Dispensary, I saw very many persons among the lower classes of society who were addicted to the intemperate use of fermented liquors, and in as many a peculiar condition of the brain supervened upon the irritable state of the stomach; and, although it could not be described as being precisely delirium tremens, yet it nearly resembled it in many of its characteristics, more especially in the watchfulness, the appearance of phantasmata during the night, and the tremulousness of the hands and tongue.

These symptoms in every case gave way to opium in conjunction with tonics. I may also state, that they all had that peculiar debility which belongs to this complaint. In all the instances which I have known, in which depletion was made use of, the pulse, although previously apparently full, and strong, has uniformly given way after the abstraction of even a very few ounces of blood, and has never again rallied. I can therefore only suppose, that the disease called in Boston Delirium Tremens, is not the same as the one we meet with in this country.

The case I am about to relate is neither singular in its symptoms, nor different from those of other persons who have suffered similar attacks; but merely serves to shew the utility of perseverance in a remedy when once you have determined that you are right in your diagnosis; and further, the benefit which may be expected from a steady continuance in the use of opium, in such a state of cerebral excitement.

On the 19th August, 1834, I was requested to see J. L., æt. 33, of a leucophlegmatic habit, and who had previously suffered from an attack of apoplexy, from which he recovered by the usual means. I found him labouring under delirium tremens, which had commenced two days before, in the night, having been accompanied by an epileptic paroxysm. He was enjoined quietude both by myself and by my friend, Mr. Benman, but he would not listen to us, and expressed extreme anxiety to go a short distance from town, where he had just embarked in an extensive concern. We both thought that he had better remain quiet at home, but he very much insisted that he would not; and, as he had an establishment at the house he wished to visit, we reluctantly permitted him to go.

The next morning we found that he had had another epileptic attack, and had been very restless during the night, notwithstanding we had augmented the dose of opium. He now remained at home, and we placed him under control, rather than restraint, although at intervals the latter was absolutely necessary. We continued to increase the quantity of opium both in a solid form, and also in that of tincture, until the 23d, when, on the evening of that day, he had nine hours of uninterrupted sleep, from which he awoke, if not completely cured of his tremulousness and hallucination, at least, very little of them remained: from this time his cure went on progressively. He was allowed during the attack strong beef-tea, jellies, and about ℥xx of bottled porter or stout, in the twenty-four hours, which he sometimes rather exceeded. The draughts consisted principally of Carb. Ammon. Conf. Piper. Nig. et Inf. Caryophyll. et Aq. Pimentæ. The Tinct. Opii was increased or diminished, as necessity demanded.

As I have not thought it necessary to detail each day's symptoms seriatim, I have added an account of the quantity of opium taken by this patient every day. I may here remark, that the opium never caused constipation, and that his pulse during the disease never was less than 130 in a minute, and was of a very feeble and tremulous character. As the disease abated, the Opium and Carb. Ammon. were abstracted gradually from his draughts, and Sulph. of Quinia added.

| 1834. | Tinct. Opii. | Solid Opium. |
|---|---|---|
| August. | No. of *m*. | No. of Grains. |
| 18 | 180 | |
| 19 | 120 | 2 |
| 20 | 180 | 12 |
| 21 | 320 | 24½ |
| 22 | 240 | 30 |
| 23 | 280 | 35 |
| 24 | 160 | 6 |
| 25 | 200 | 5 |
| 26 | 240 | 5 |
| 27 | 160 | |
| 28 | 120 | |
| 29 | 90 | |
| 30 | 90 | |
| 13 days | 2380*m* | 119½ |

Dividing the 2380 minims of Tinct. of Opium by 19,* will give $125\frac{5}{19}$ as the number of grains of solid opium said to be dissolved in that quantity of laudanum, which, added to $119\frac{1}{2}$, gives $244\frac{29}{38}$ grains, as the amount of the drug used in 13 days.

* Nineteen minims of Tinct. Opii contain one grain of the drug.

31, *New Bridge Street;*
*Sept.* 20, 1834.

